# Physiological and Biochemical Responses of Pearl Millet (*Pennisetum glaucum* L.) Seedlings Exposed to Silver Nitrate (AgNO_3_) and Silver Nanoparticles (AgNPs)

**DOI:** 10.3390/ijerph16132261

**Published:** 2019-06-26

**Authors:** Imran Khan, Muhammad Ali Raza, Muhammad Hayder Bin Khalid, Samrah Afzal Awan, Naveed Iqbal Raja, Xinquan Zhang, Sun Min, Bing Chao Wu, Muhammad Jawad Hassan, Linkai Huang

**Affiliations:** 1 Department of Grassland Science, Animal Science and Technology College, Sichuan Agricultural University, Chengdu 611130, China; 2 College of Agronomy, Sichuan Agricultural University, Chengdu 611130, China; 3 Maize Research Institute, Sichuan Agricultural University, Chengdu 611130, China; 4 Department of Botany, PMAS-Arid Agriculture University, Rawalpindi 46000, Pakistan

**Keywords:** comprehensive, accumulation, antioxidants, photosynthesis, phytotoxic

## Abstract

A rapid and continuous growth of silver nanoparticles (AgNPs) via their precursor “silver nitrate” (AgNO_3_) has increased their environmental risk because of their unsafe discharge into the surrounding environment. Both have damaging effects on plants and induce oxidative stress. In the present study, differential responses in the morpho-physiological and biochemical profiles of *P. glaucum* (L.) seedlings exposed to various doses of AgNPs and AgNO_3_ were studied. Both have forms of Ag accelerated the reactive oxygen species (ROS) production, which adversely affected the membrane stability as a result of their enhanced accumulation, and resulted in a significant reduction in growth, that is, root length, shoot length, fresh and dry biomass, and relative water content. AgNO_3_ possessed a higher degree of toxicity owing to its higher accumulation than AgNPs, and induced changes in the antioxidants’ enzyme activity: superoxide dismutase (SOD), peroxidase (POD), catalases (CAT), guaiacol peroxidase (GPX), ascorbate peroxidase (APX), and glutathione reductase (GR) activity, as well as proline content, total phenolic, and total flavonoids contents (TFCs) under all tested treatments (mM). A decline in photosynthetic pigments such as total chlorophyll content and carotenoid content and alterations in quantum yield (Fv/Fm), photochemical (qP), and non-photochemical quenching (NPQ) indicated the blockage of the electron transport chain (ETC), which led to a significant inhibition of photosynthesis. Interestingly, seedlings exposed to AgNPs showed less damaging effects on *P. glaucum* (L.) seedlings, resulting in relatively lower oxidative stress in contrast to AgNO_3_. Our results revealed that AgNO_3_ and AgNPs possessed differential phytotoxic effects on *P. glaucum* (L.) seedlings, including their mechanism of uptake, translocation, and action. The present findings may be useful in phytotoxic research to design strategies that minimize the adverse effects of AgNPs and AgNO_3_ on crops, especially in the agriculture sector.

## 1. Introduction

Pearl millet (*P.glaucum* L.) is one of the premium and important food crops that occupy the sixth rank in the world, with a huge cultivated area (60%) in Africa and (35%) in Asia. It covers about half of the total global production of millets and is utilized as a staple food, source of protein for human beings, and fodder for livestock [1,2]. Pearl millet grains are used to make flour, bread, pasture, and “coucous” [3]. As livestock fodder, it is mostly grown to produce hay, green-chop, silage, pasture, and stands over feed grazed directly [4,5]. The seedling stage of plants is very sensitive to different types of stresses, which leads to a great loss of crop production in agriculture system. Keeping in view the world human’s population is prophesied to touch 9.6 billion by 2050, from today’s total of about 7 billion [6], it will be the staple food for human beings in the coming years and extensively utilized by animals as feedstuff.

Nanotechnology is the science that relates to nanomaterials that possess molecular and atomic dimensions of less than a few nanometers. In recent years, most of the research work has been performed to explore the effects of nanoparticles (NPs) on plants, including other living organisms [7]. NPs are extensively being used in physics, chemistry, agricultural science, environmental science, and medicine [8], but their interactions with plant metabolism still needed more attention. Some different and contradictory assays have been reported on the applications of NPs with reference to absorption, uptake, accumulation, transformation, and their effects in fewer plant species [9]. After reduction, the ionic material from industrial discharge forms clusters and changes into nanoparticles, which may be taken up by plants through different ways [10]. Proper regulation and the safe discharge of industrial impurities into soil and water to control their adversative effect on plants requires further effort and investigation [11].

**Figure 1:**
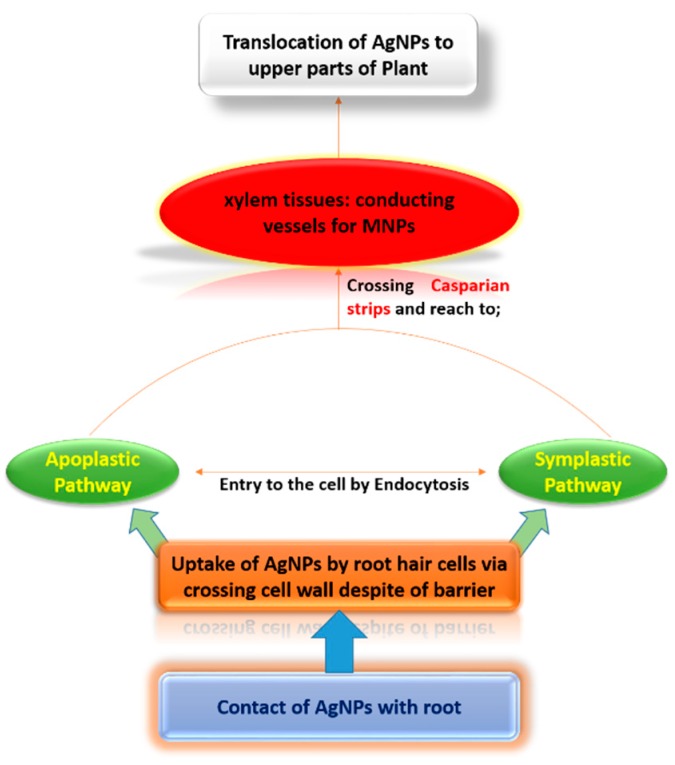
A possible mechanism of uptake and distribution of AgNPs in plants.

Various chemical compounds (chemical fertilizers and minerals) have been tested in agricultural systems to improve crop production, but unfortunately, they cause some serious health issues for human beings and side effects on plants, and harm the environment and soil in different ways. It has been found that less functional toxic elements in the biological systems make their way into the food chain, accumulate in plant tissues (Figure 1), and cause lethal effects to plant species [12]. In agriculture, metal-based NPs such as zinc (Zn), silver (Ag), copper (Cu), iron (Fe), and titanium dioxide (TiO_2_) have been tested for crop improvement, but they induce more toxicity to crop plants. Among them, the interaction of AgNPs with plants is being tested on a large scale in the nano-research field [13]. Phytotoxic effects of AgNPs on plants depend upon the age and type of plant species, sizes and concentrations of nanoparticles, experimental conditions, and the duration of the experiment. Reported investigations have shown that AgNPs release Ag^+^ into the surrounding environment, which leads to inhibition of respiratory enzymes and ultimately causes oxidative stress by producing reactive oxygen species (ROS) [14]. The main causes of toxic effects of AgNPs are still unclear, but may be caused by silver ions or their intrinsic properties. Existing literature on the phytotoxic effects of AgNPs is still limited and it is more important to explore their phytotoxic effects, as their productive and destructive effects on the environment are not fully known.

Moreover, it has been reported that as the concentration of AgNPs increases in plants, a reduction in root and shoot length and biomass strongly directs the toxicity to increase in plants [15]. The authors of [16] also reported that the interaction of NPs with plants had significant impacts on seed germination and seedling growth, and was dependent on the concentrations and properties of NPs and plant species. The accumulation of AgNPs in plant cells seems to be dependent on system reduction potential, and as such, NPs have both constructive and damaging impacts on the germination of seeds and root growth [17].

**Figure 2:**
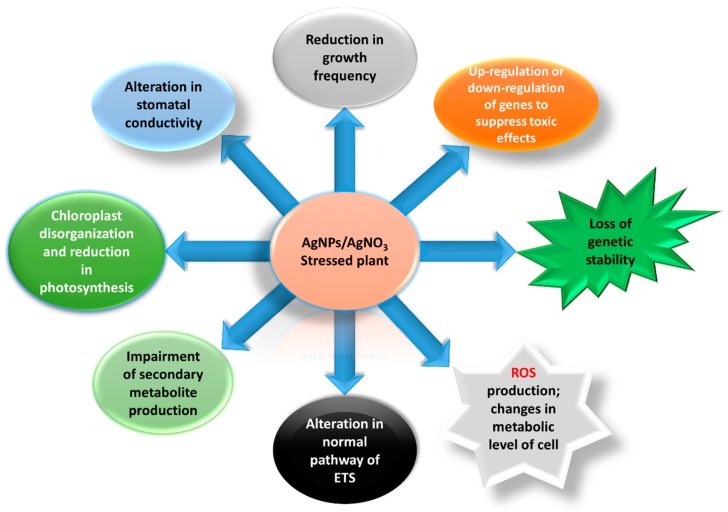
An overview of damaging effects due silver nitrate (AgNO_3_) and synthesized metallic silver nanoparticles (AgNPs).

In addition, the phytotoxic effects of silver nitrate (AgNO_3_) are also less explored in different plant species [18]. The release of silver ions from AgNO_3_ causes severe toxicity to a variety of organisms such as plants, algae, animals, and bacteria as in (Figure 2) and is based on their inhibitory potential [19]. Published literature has demonstrated more hazardous and toxic effects of AgNO_3_ on fruiting, flowering, and other physiological mechanisms of plants, which reflects a threat to sustainable agriculture around the world [20]. There are just a few reports that have described the effect of AgNPs on the morphological parameters of pearl millet, while no report has explained the phytotoxic effect of AgNO_3_ on the morphological, physiological, or biochemical profiles of pearl millet. Therefore, the current study investigated the toxicity levels of AgNPs and AgNO_3_ at different concentrations on *P. glaucum* by analyzing seedling growth, absorption, accumulation, oxidative stress, and antioxidant enzyme activity, which were still unknown.

## 2. Materials and Methods

### 2.1. Sliver Nanoparticles and Silver Nitrate

**Figure 3:**
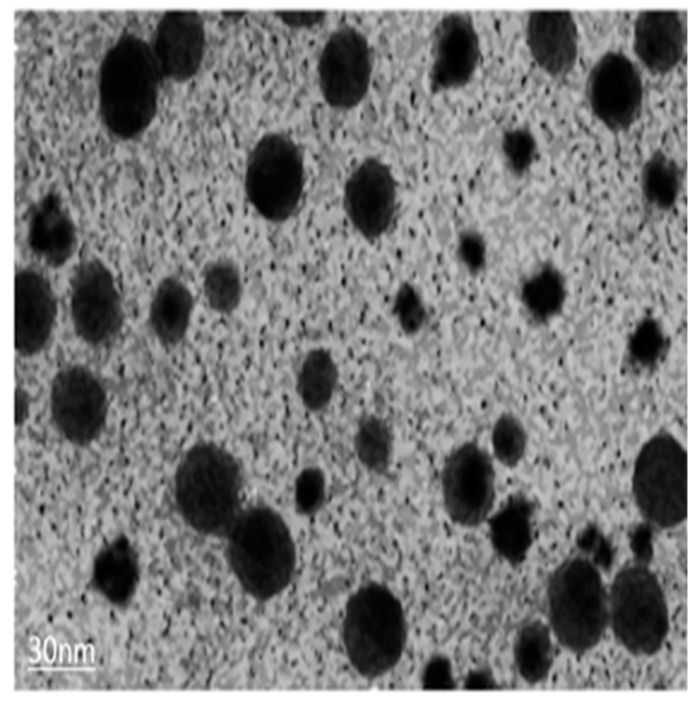
Transmission Electron Microscopy (TEM) of synthesized metallic Silver nanoparticles AgNPs.

In the present study, silver nitrate and synthesized silver nanoparticles (AgNPs) were obtained from NANOCS (Nanocs lnc. New York, NY, USA), manufactured according to >0.75A520 units/m having 30 nm size (solution form) as shown in (Figure 3). The dilution of nanoparticles was taken out using stock solution.

### 2.2. Plant Material and Sterilization

The seeds of *Pennisetum glaucum* were used as an explants for this experiment. Seeds were washed with simple tap water, dipped in ethanol for thirty minutes, then sterilized in 10% (v/v) sodium hypochlorite solution (NaOCl) for 10 min, and again washed with autoclaved distilled water. To break dormancy, seeds were soaked in double distilled water for 12 h and then transferred to a cotton cloth for germination. Uniform sized vigorous seeds were placed in petri plates lined with filter paper containing half strength Hoagland solution and allowed to germinate at 25 ± 2 °C for four days in the dark. After germination, five seedlings with almost same length were again placed in half strength Hoagland solution (40 mL) per pot (6 cm × 6 cm); placed in a growth chamber at 28 ± 2 °C under 11:13 h dark and light periods with light intensity of 200,000 lux; and exposed to AgNO_3_ and AgNP treatments for 24 h at a dosage of 2, 4, and 6 mM along with a control.

### 2.3. Growth Parameters

To assess the growth, ten seedlings from each treatment including the control were randomly selected and their fresh mass was weighed. Root and shoot length was measured using a centimeter scale and root and shoot fresh mass was measured following the method provided by the authors of [21]. The relative water content (RWC) of the leaf was estimated as (FW − DW)/(TW − DW) × 100, where FW fresh weight of leaf tissues, DW dry weight of leaf tissues, and TW turgid weight of leaf after equilibration in distilled water for 24 h.

### 2.4. Estimation of Photosynthetic Pigments

The estimation of total chlorophyll content, fresh leaf samples (20 mg) were taken from the seedlings (control and treated samples). Leaves were grounded and homogenized in 80% acetone followed by pigment extraction and centrifugation. The absorbance of mixture was measured at 663 nm and 646 nm using a spectrophotometer, and chlorophyll content was assessed by following the protocol described by the authors of [22].

### 2.5. Biochemical Profiling

#### 2.5.1. Hydrogen Peroxide Content and Lipid Peroxidation

Estimation of hydrogen peroxide (H_2_O_2_) as ROS in the seedlings was carried out by ferrithiocyanate method, provided by the authors of [23]. The absorbance of samples was measured at 480 nm and the content of H_2_O_2_ in every sample was calculated using a standard curve.

Lipid peroxidation as malondialdehyde (MDA) was estimated according to the protocol from the work of [24] with some modifications and absorbance was recorded at 532 nm, whereas the non-specific absorbance at 600 nm was subtracted. Calculation of recorded MDA content was carried out with an extinction coefficient of 155 mM cm^−1^.

#### 2.5.2. Proline Content

Proline content was measured using protocol from the work of [25]. The plant samples were homogenized with a pestle and mortar in 5 mL of 3% sulphosalycylic acid. Ninhydrin reagent (2 mL) and glacial acetic acid (2 mL) were added to the test tube with 2 mL of extract. The mixture was placed in a water bath and boiled at 100 °C for thirty minutes. Then, 6 mL of toluene was added to reaction mixture after cooling and was transferred to a separate funnel. Thoroughly mixing resulted in the separation of chromophore with toluene and absorbance was measured at 520 nm using a spectrophotometer.

#### 2.5.3. Superoxide Dismutase (SOD) Activity

One of the methods described by the authors of [26] with some modifications was used to measure the SOD activity. One milliliter of reaction mixture was composed of 1 mM Ethylenediamine tetraacetic acid (EDTA), 130 mM methionine, 0.05 molar phosphate buffer (pH 7), 0.02 mM riboflavin, and 0.75 mM nitroblue tetrazolium (NBT). The reaction mixture was placed under the fluorescent light for seven minutes and absorbance was measured at 560 nm. SOD activity was calculated by following the Lambert–Beer law equation:

A = εLC,(1)

where A is the absorbance, ε is the extinction coefficient, L is the length of each wall, and C is the concentration of enzymes.

#### 2.5.4. Estimation of Catalases, Peroxidases, Ascorbate Peroxidase Activities, and Protein Contents

The protocol from the work of [27] was used with some modifications to determine the catalase activity. Enzyme extract (0.5 mL) was added to 3 mL of the reaction mixture (50 mM phosphate buffer, pH 7.0 and 30% w/v H_2_O_2_). The catalase activity was examined at the decrease of absorbance at 240 nm.

The estimation of peroxidase activity was measured by following the method from the work of [28]. Then, 3.0 mL of the reaction mixture containing 20 mM guaiacol, 10 mM phosphate buffer, and 10 mM H_2_O_2_ was mixed with 0.5 mL of enzyme extract (heated in water bath at 45 °C for five minutes before mixing). An increase in absorbance was measured at 470 nm due to the formation of tetraguaiacol [29].

Ascorbate peroxidase (APX) activity was performed according to the protocols from the work of [30]. Determination of protein contents in each sample were carried out according to protocols from the work of [31].

#### 2.5.5. Glutathion Reductase (GR) and Guaiacol Peroxidase (GPX) Activity

GR activity was determined according to methods from the work of [32] by measuring the increase in absorbance range due to the presence of 5,5-dithiobis-2-nitrobenzoic acid (DTNB) and oxidized glutathione. GR activity (one unit) might be referred to as the quantity of enzyme needed to cause oxidation of NADPH (1.0 μm) at neutral pH (7.5).

GPX activity was assessed by the method from the authors of [28] by evaluating the formation of tetra guaiacol at 470 nm. One unit of GPX was the amount required to catalyze the conversion of H_2_O_2_ (1.0 μmol) per min.

#### 2.5.6. Estimation of Total Phenolic Content (TPC)

For TPC estimation, Folin–Ciocalteu reagent (0.75 mL) was added to 100 µl plant extract, gently mixed, and placed them at 22 °C for five minutes. Then, 0.75 mL of Na_2_CO_3_ solution was added to the mixture and kept at 22 °C for ninety minutes. The final results were concluded by checking the sample absorbance at 725 nm with a UV/vis-DAD spectrophotometer [33].

#### 2.5.7. Estimation of Total Flavonoids Content (TFC)

For the estimation of TFC, an AICI_3_–NaNO_2_-NaOH reaction complex was used according to protocol given by the authors of [34]. A total of 0.2 mL extract was added to 3.5 mL distilled water. Furthermore, (0.15 mL) 5% NaNO_2_, (0.15 mL) 10% AlCl_3_, and (1 mL) 1M NaOH were added to the mixtureat equal 5 min time intervals, and placed at normal room temperature for 15 min. Reaction absorbance was measured at 510 nm using a UV/vis-DAD spectrophotometer.

### 2.6. Experimental Design and Statistical Analysis

Experiments were performed twice with three replicates (*n* = 6). Means and standard errors (±) were calculated by analysis of variance (one-way). The comparison of the means (control + treatments) was confirmed by Duncan’s multiple range test at *p* < 0.05.

## 3. Results

### 3.1. Growth Parameters

The induced impact of AgNPs and AgNO_3_ on the seedlings’ growth of *P.glaucum* was analyzed and the results are presented in Table 1. Root length, shoot length, and fresh and dry biomass were measured to evaluate the phytotoxic effects of AgNPs and AgNO_3_ on the growth of *P.glaucum* seedlings. The results indicated that AgNO_3_ significantly (*p* < 0.05) reduced the root and shoot length with the increasing concentration (2 mM, 4 mM, 6 mM), while AgNPs showed a minor reduction when both were compared with the control. Root and shoot length were reduced to 41% and 21% at 2 mM AgNO_3_, whereas AgNPs under the same treatment were noted to cause a reduction of 28% and 15% for root and shoot length, respectively (Table 1), which indicated a smaller decrease in growth parameters at a lower concentration. However, a significant reduction was observed at a higher concentration (6 mM) of AgNO_3_ with a 68% and 36% in root and shoots length when compared with AgNPs, which demonstrated a reduction of 61% and 31% in root and shoot, respectively, lesser than the AgNO_3_. Fresh and dry biomass of seedlings exposed to AgNO_3_ and AgNPs was also observed to be lower than control. AgNO_3_ (2 mM) reduced the fresh and dry biomass of seedlings by 38% and 20%, respectively, while on the other hand, AgNPs at same concentration caused a reduction in fresh and dry biomass only by 14% and 10%, respectively. A greater reduction in biomass was recorded with increasing the concentration of both AgNO_3_ and AgNPs. A higher concentration (6 mM) of AgNPs reduced the fresh and dry biomass of seedlings by up to 47% and 35%, respectively, while the reduction was 55% for fresh biomass and 45% for dry biomass with 6 mM AgNO_3_. Compared with the control, the RWC was gradually decreased to 28% and 26% at 2 mM for AgNO_3_ and AgNPs, whereas a further decline in RWC was noted under all treatments of AgNO_3_ than AgNPs (Table 1). These presented results indicated that AgNO_3_ possessed more damaging effects on growth parameters of *P.glaucum* than AgNPs.

### 3.2. Photosynthetic Pigments and Protein Contents

**Figure 4:**
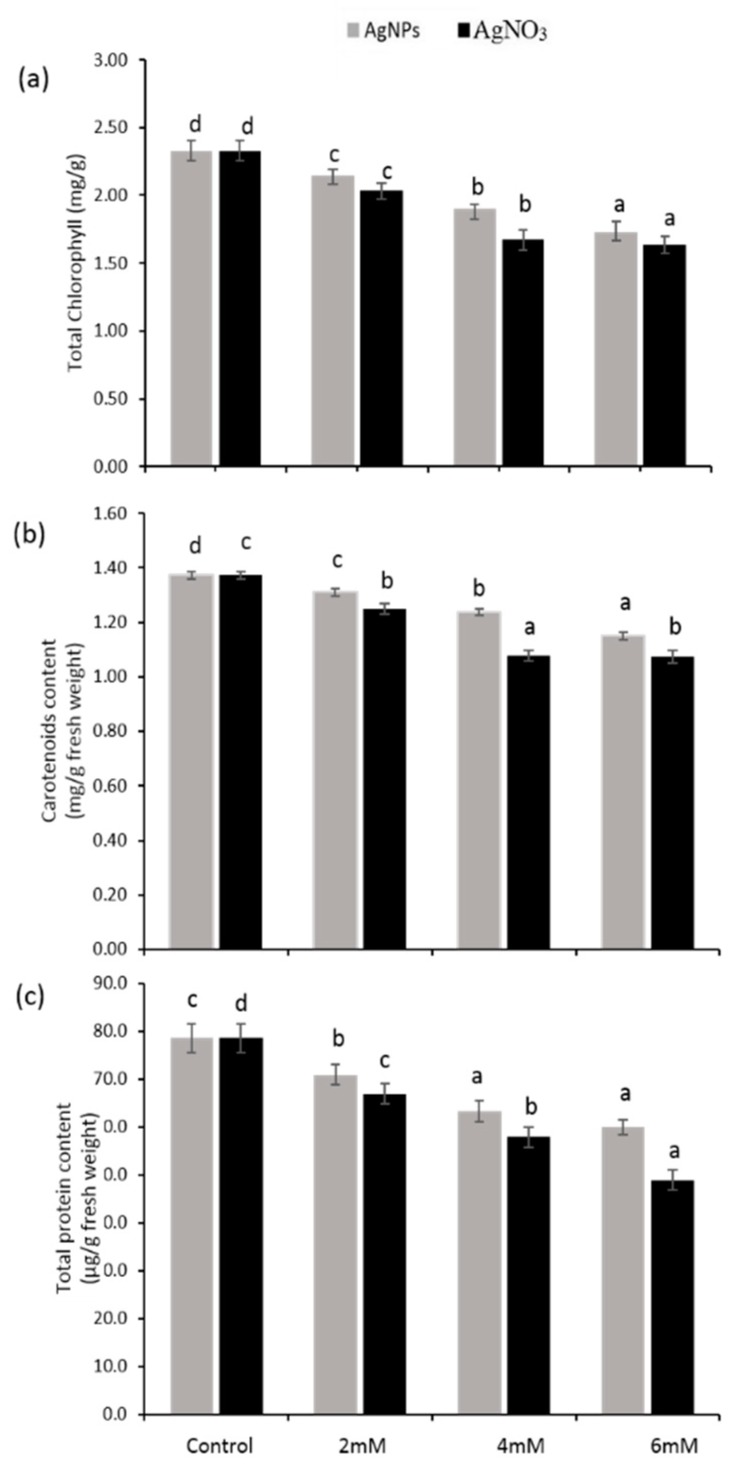
Total chlorophyll content (**a**), carotenoids content (**b**) and total protein content (**c**) contents in the seedlings of *P. glaucum* L. exposed to AgNPs and AgNO_3_. The Experiment was performed twice with three replicates (*n* = 6). Means and standard errors (±) were calculated by analysis of variance (one way). A comparison of the means (control + treatments) was confirmed by Duncan’s Multiple Range Test at *p* < 0.05.

Total chlorophyll and carotenoid content decreased under all treatments (2 mM, 4 mM, and 6 mM) of AgNO_3_ and AgNPs, whereas the effect of AgNPs at the similar dose was slightly lesser when compared with the control (Figure 4a,b). It was obvious that the trend of reduction in carotenoids was less than the chlorophyll for all treatments. However, the total protein content in *P. glaucum* seedlings exposed to various doses of AgNO_3_ and AgNPs seemed to be less than the control (Figure 1c). At a high concentration of AgNO_3_ (6 mM), significant reduction was recorded (38%) with respect to AgNPs at the same dose (24%) when compared with the control. Furthermore, at 2 mM of AgNO_3_ and AgNPs, a reduction in the percentage of total protein content of 15% and 9%, respectively, was noticed. These results clearly show that AgNO_3_ had a more destructive effect than AgNPs.

**Figure 5:**
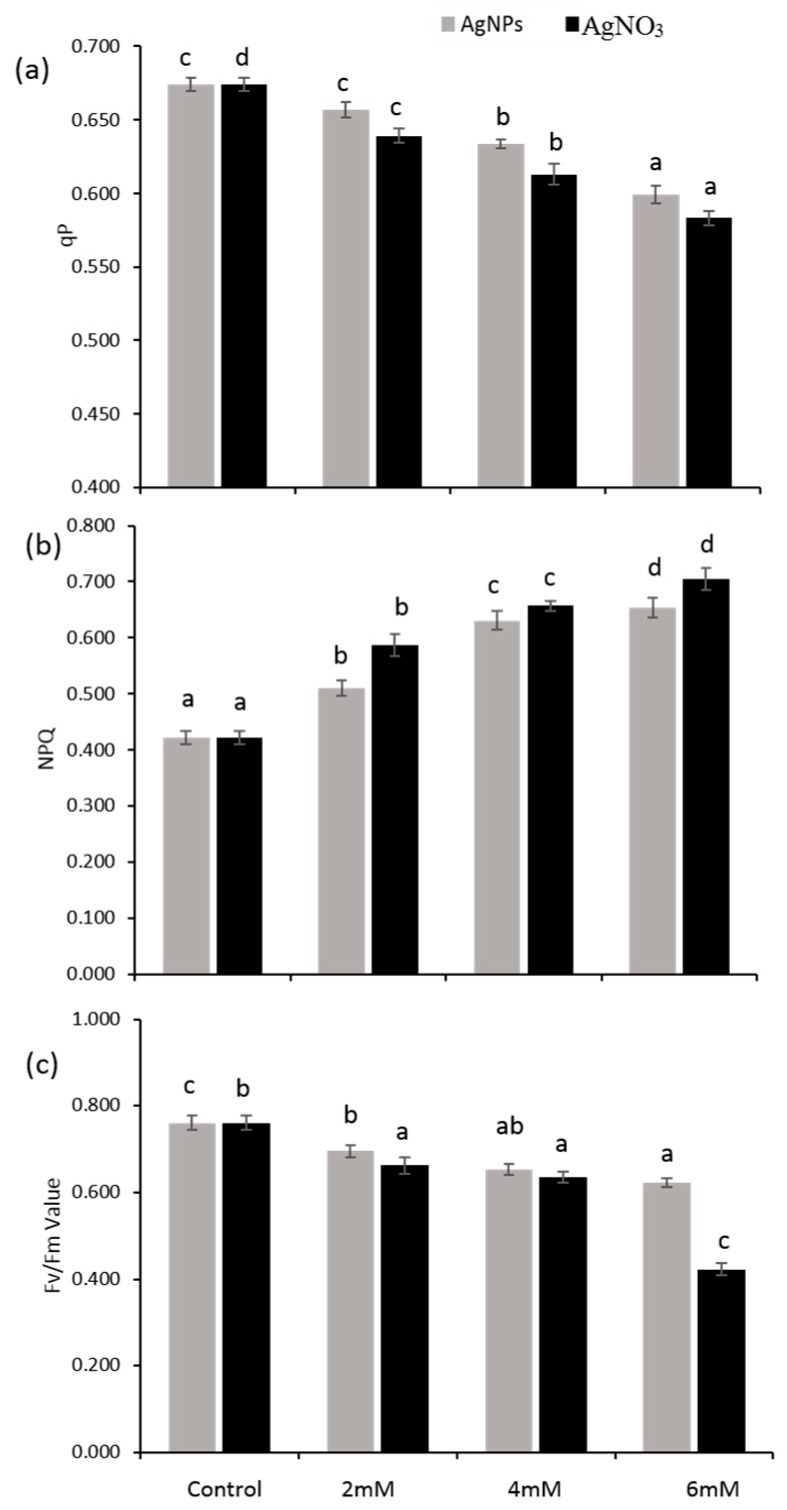
Photochemical quenching (**a**), NPQ, non-photochemical quenching (**b**) and Fv/Fm values (**c**) of *P. glaucum* L. seedlings exposed to AgNPs and AgNO_3_. The Experiment was performed twice with three replicates (*n* = 6). Means and standard errors (±) were calculated by analysis of variance (one way). And the A comparison of the means (control + treatments) was confirmed by Duncan’s Multiple Range Test at *p* < 0.05.

Chlorophyll fluorescence provided the state of health for the photosynthetic system in the leaves. A significant decrease in Fv/Fm value was recorded when seedlings were exposed to AgNO_3_ as compared with the control (Figure 5c). AgNO_3_ at the concentrations (2 mM, 4 mM, and 6 mM) strongly influenced the photosynthetic performance of *P. glaucum* seedlings when compared with the AgNPs and the control. Figure 5a,b shows a lower value for photochemical quenching (qP), but a high value for non-photochemical quenching (NPQ) was observed in the seedlings exposed to given treatments.

### 3.3. Oxidative Damage

**Figure 6:**
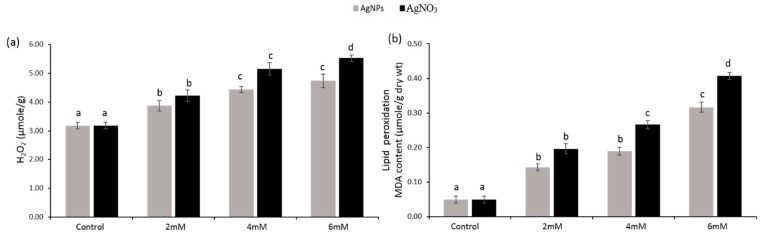
Hydrogen peroxide (H_2_O_2_) (**a**) lipid peroxidation (**b**) as melondialdehyd (MDA) content in the seedlings of *P. glaucum* L. exposed to AgNPs and AgNO_3_. Experiments were performed twice with three replicates (*n* = 6). Means and standard errors (±) were calculated by analysis of variance (one-way). A comparison of the means (control + treatments) was confirmed by Duncan’s multiple range test at *p* < 0.05.

The results pertaining to hydrogen peroxide (H_2_O_2_) contents in the seedlings of pearl millet exposed to variable concentration of AgNO_3_ and AgNPs are presented in Figure 6a. Compared with the control, silver nitrate- and silver nanoparticles-treated seedlings significantly (*p* < 0.05) enhanced the hydrogen peroxide accumulation, while AgNO_3_-treated seedlings exhibited a higher H_2_O_2_ level than AgNPs.

### 3.4. Lipid Peroxidation as MDA Contents

The data shown in Figure 6b indicate that AgNO_3_ and AgNPs caused significant damage to the cellular membrane as melondialdehyd (MDA) contents, the value of lipid peroxidation was progressively raised by increasing the concentration of silver nitrate and silver nanoparticles (2 mM, 4 mM, and 6 mM). AgNO_3_-treated seedlings at 6 mM showed significantly (*p* < 0.05) higher accumulation of MDA (84.9 µM/g dry wt) than AgNPs seedlings (72.7 µM/g dry wt) and control seedlings (23.2 µM/g dry wt). While on the other hand, MDA content in AgNO_3_ and AgNPs treated seedlings at 2 mM was (46.5 µM/g dry wt) and (35.7 µM/g dry wt) respectively.

### 3.5. Enzymatic Antioxidants

**Figure 7:**
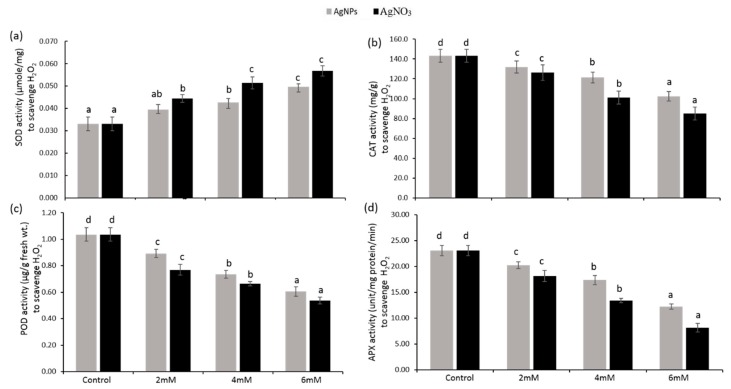
Activity of superoxide dismutase (SOD) (**a**), catalase (CAT) (**b**), peroxidase (POD) (**c**), and ascorbate peroxidase (APX) (**d**) in *P. glaucum* L. seedlings exposed to AgNPs and AgNO_3_. Experiments were performed twice with three replicates (*n* = 6). Means and standard errors (±) were calculated by analysis of variance (one-way). A comparison of the means (control + treatments) was confirmed by Duncan’s multiple range test at *p* < 0.05.

**Figure 8:**
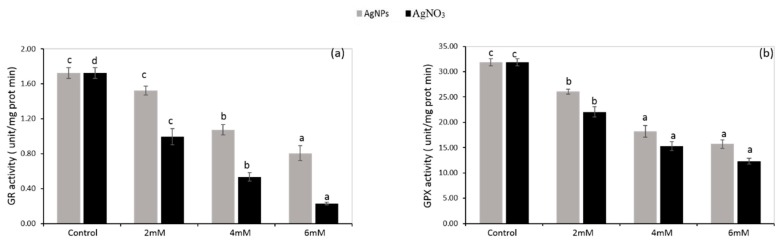
Glutathione reductase activity (a) and guaiacole peroxidase activity (b) in the *P. glaucum* L. seedlings exposed to AgNPs and AgNO_3_. Experiments were performed twice with three replicates (*n* = 6). Means and standard errors (±) were calculated by analysis of variance (one way). A comparison of the means (control + treatments) was confirmed by Duncan’s multiple range test at *p* < 0.05.

The results related to enzymatic antioxidants such as superoxide dismutase (SOD), ascorbate peroxidase (APX), catalase (CAT), peroxidase (POD), guaiacol peroxidase (GPX), and glutathion reductase (GR) are presented in Figure 7 and Figure 8. SOD activity in both treated seedlings exposed to AgNO_3_ and AgNPs at a 2 mM dose exhibited a higher rate (25% and 15%, respectively) when compared with the control. As their concentration increased to 6 mM, a progressive and significant (*p* < 0.05) trend was recorded that boosted the SOD activity up to 42% for AgNO_3_ and 34% for AgNPs (Figure 7a). Further decline in SOD activity was noticed in the seedlings following AgNO_3_ contact when compared with AgNPs, which disclosed more harmful impact of silver nitrate on *P.glaucum*. Ascorbate peroxidase (APX) is highly responsible for disassociating hydrogen peroxide into H_2_O and oxygen using ascorbate (electron donor), whereas CAT dissociates hydrogen peroxide into H_2_O and oxygen without using any external reductants. Significant (*p* < 0.05) inhibition of CAT and APX activity was noted in *P. glaucum* seedlings with respect to the increase in AgNO_3_ and AgNPs dose (6 mM) up to 64% and 47%, and 29% and 41%, respectively, by comparing the control plants. The rate of inhibition in their activities under AgNPs treatment was recorded to be less than silver nitrate, as presented in Figure 7b,d.

*P. glaucum* seedlings under AgNO_3_ and AgNPs treatment (2 mM) exhibited lower peroxidase (POD), glutathione reductase (GR), and guaiacol peroxidase (GPX) activity (26%, 42%, and 31%, respectively; and 14%, 12%, and 18%, respectively), while their activity was gradually decreased up to 48%, 86%, and 61%, and 41%, 53%, and 51%, respectively, by increasing the concentration (6 mM) of silver nitrate and silver nanoparticles, respectively. High doses of both caused a down-regulating effect on POD, GR, and GPX activities, which showed a significant (*p* < 0.05) reduction, but their level was less under AgNPs than AgNO_3_ when compared with the control (Figure 7c and Figure 8a,b ).

### 3.6. Non-Enzymatic Antioxidants

Table 2 shows the response of non-enzymatic antioxidants such as proline contents, and total phenolic and flavonoid content under different concentrations of AgNO_3_ and AgNPs. Control seedlings exhibited relatively lower contents of non-enzymatic antioxidants, while their levels increased as the doses of AgNO_3_ and AgNPs increased. For all treatments of AgNO_3_ and AgNPs, proline content was recorded to be less than the control, whereas others (TPCs and TFCs) showed a progressive effect. An enhancing trend of non-enzymatic antioxidants seemed to be greater in *P.glaucum* seedlings exposed to 5mM of AgNO_3_ than AgNPs, presenting proline (44%, 41%), TPCs (38%, 33%), and TFCs (52%, 48%), respectively when compared with control. Levels of non-enzymatic antioxidants were significantly (*p* < 0.05) elevated with respect to the exposure of both AgNO_3_ and AgNPs compared with the control, but the higher activity was attributable to AgNO_3_, as shown in Table 2.

## 4. Discussion

The current study was carried out to examine the phytotoxic impact of AgNO_3_ and AgNPs on *P.glaucum* seedlings. A significant decrease in growth parameters in *P.glaucum* seedlings exposed to variable doses of AgNO_3_ and AgNPs was presented (Table 1). Moreover, stunted plant growth, short leaf length, and distortion of shoots were also analyzed, which might have a direct link with the poor photosynthetic performance of the seedlings. The toxic impact of AgNPs on root length, shoot length, fresh and dry biomass, photosynthetic pigments, and biochemical profiles was observed to be less for AgNO_3_ when both were compared with controls. A similar study was reported by the authors of [35], where AgNO_3_ severely reduced the growth parameters by increasing the uptake of silver in plants. More accumulation of silver from AgNO_3_ caused inhibition of grain germination, reduction in root and shoot length, and a decrease in the chlorophyll pigments in barley seedlings [36]. However, AgNPs possessed no more toxic effect on the plant morphology and increased the cell division process by enhancing the hormonal activities, dependent on the size of the NPs [37]. The published literature suggests that the response to Ag^+^ (bulk or nano) may be positive or negative, particularly depending on the interaction of proteins with the plant internal metabolism. Although AgNPs inhibited the root elongation in corn, their application also enhanced the morphological characteristics including seed germination, root and shoot length, and biomass in watermelon and cucumber [10,38]. Furthermore, alterations in root and shoot length were also determined from the time of germination, which might be the result of contact with AgNPs, which may create ‘nano holes’ in the seed coats and make their entry easier to seeds via the seed coat, resulting in enhanced germination. The slow and slight release of silver ions (Ag^+^) could be a second major reason that Ag-nanoparticles have no deleterious effects. Penetration, accumulation, and translocation of AgNPs appears to be strongly dependent on their size, shape, concentration, and type of plant species [37]. Our results reaffirmed those of the authors of [39], who described AgNO_3_ as more toxic to seedlings’ growth and morphology. Interestingly, in the present study, no more harmful effects of AgNPs on *P.glaucum* seedlings were observed even at a high concentration; these findings strongly correlate with those of the authors of [40], who reported high germination rate and growth parameters in *R.cummunis* exposed to higher doses of AgNPs.

In the present study, seedlings exposed to AgNPs had a minor reduction in root and shoot length (Table 1). However, few reports have described that nano silver improved the water uptake through accumulation, and transportation of Ag^+^ in roots caused distortion of epidermal structures and changed the anatomical features of plants [41]. Further distortion of roots and damage to roots were observed in the seedlings treated with AgNO_3_, while AgNPs treatment did not behave like AgNO_3_ treatment. Our study strongly matches with previous findings that more silver ions in roots alter its structure and also associate with translocation mechanism in plants [42].

Photosynthetic parameters can be easily measured by estimating chlorophyll fluorescence under stress and normal growth conditions [42]. The results showed a significant reduction in total chlorophyll (Figure 4a) dependent on the decrease in Fv/Fm and qP values in *P.glaucum* seedlings exposed to AgNO_3_, whereas a lower reduction in photosynthetic parameters was noticed in the case of AgNPs-treated seedlings when both were compared with the control (Figure 2a,b). Higher chlorophyll content and high efficiency of photosynthetic system was reported in *Brassica* seedlings exposed to AgNPs [43]. Various stresses caused more reduction in the activity of photosystem II, which ultimately decreased the Fv/Fm and qP value and alternatively declined the plant chlorophyll content [44]. Our results demonstrated that a decline in the Fv/Fm and qP value (Figure 5a,c) resulted in a decreased chlorophyll content, which was strongly associated with lower biomass accumulation in *P. glaucum* seedlings. Previous data showed that NPQ values increased when plants were facing stress conditions, which led to the down-regulation of photosystem II and inhibited the functioning of the electron transport chain (ETC) [45]. Present findings demonstrated high values of NPQ in the seedlings exposed to AgNO_3_, but lower in AgNPs, which indicates proper functioning of the ETC in the seedlings treated with silver nanoparticles (Figure 5b). The present results demonstrated that more ROS production decreased the seedlings’ growth and caused protein oxidation and lipid peroxidation. Damaging effects of AgNPs on oxidative stress seemed to be lower with AgNO_3_ when compared with the control. A recent study reported that a decline in growth attributes might be the result of destructive impacts of ROS on the photosynthetic machinery and may be involved in oxidative stress [14]. Previously, it was described that stress condition triggers the production of ROS and accelerates the oxidative damage [46]. To cope with oxidative stress inside the cells, plants have a well-developed antioxidant defense system.

A significant (*p* < 0.05) reduction in enzymatic antioxidants such as APX (Figure 7d), CAT (Figure 7b), POD (Figure 7c), GR (Figure 8a), and GPX (Figure 8b) was recorded in the seedlings exposed to AgNO_3_ when compared with AgNPs and the control. The authors of [47] reported that CuNPs and their precursor, CuCl_2_, decreased the CAT activity as a result of the direct interaction of copper with thiol moieties of protein, and altered the CAT structure, which led to inhibition of its functioning. A similar mechanism might be regulated by Ag in *P. glaucum* seedlings, resulting in enhanced ROS production along with the suppression of enzymatic antioxidants. Previously, it was investigated that a higher concentration of AgNPs caused a reduction in CAT and APX activity in potato seedlings [48]. In another study [49], it was revealed that AgNPs had no damaging effects on wheat seedlings, though their application boosted the early growth of brassica by controlling the antioxidant capacity [43]. Exposure to stress in plants resulted in high ROS production, which affected the plant metabolism by damaging the defense mechanism and consequently decreasing the antioxidant activities [50,51]. Under stress conditions, GR plays a key role to regulate the ascorbate–glutathione cycle and converts the oxidized glutathione to glutathione [52], whereas AgNO_3_ had more toxic effects on plants related to morphology, physiology, and bioaccumulation [53]. In the current study, we observed that *P.glaucum* seedlings showed a lower activity of these antioxidants when exposed to a higher concentration of AgNO_3_ and AgNPs, but a greater reduction was found in the case of AgNO_3_.

Increased POD activity under heavy metal stress increased the growth and development of plant seedlings, which alternatively minimized the oxidative damage [54]. AgNO_3_ treatment aroused the POD activity in radish seedlings to cope with oxidative stress caused by ROS production, whereas a non-significant increase in POD activity was recorded after exposure to AgNPs [55]. Moreover, another study revealed that AgNPs increased ROS in wheat seedlings, which led to oxidative stress in plants [56]. Environmental stress led to more production of ROS and plants need to scavenge ROS for their normal growth, but stress altered the enzymatic activities involved in scavenging ROS [57]. However, in the recent study, lower POD activity was observed under higher doses of AgNO_3_ and AgNPs, which might be the result of greater ROS production and alteration in the structure of antioxidants, but a non-significant increase in ROS due to AgNPs was recorded. Our results are in line with those of the work of [58], which described that AgNPs involved in the blockage of electron transfer causes oxidative stress. Although, in the present study, *P.glaucum* seedlings exposed to AgNPs showed a reduction in CAT, APX, GPX, and POD activities when compared with the control a comparatively lower reduction was noted when compared with AgNO_3_ (Figure 7 and Figure 8), this might indicate that AgNPs have a strong interaction with proteins found in the lipid bilayer and cytosol; thus, altering its configuration and negatively influencing the antioxidant define systems [59]. Previously, it has been reported that the impact of AgNPs on antioxidant enzymes varied with plant species, dosage, and time duration of AgNPs applied [60].

In addition, plants’ cells also contain a variety of non-enzymatic antioxidants such as proline, flavonoids, and phenolic contents to mitigate the toxic effects of ROS. Previously published literature has revealed that proline content was increased when plants were exposed to various types of stresses [61], and the same trend was noted in the current study (Table 2). A significant (*p* < 0.05) increase in proline contents was recorded in the seedlings exposed to a higher concentration of AgNO_3_, which reflected a greater stress condition. Accumulation of proline to a higher level under stress indicates the that proline as a cytoplasmic osmolyte protects the protein against denaturation [62]

Seedlings treated with AgNO_3_ showed higher levels of TPC and TFC when compared with both AgNPs and controls; AgNPs treatment also considerably enhanced the TPC and TFC levels, but possessed less toxic effects than AgNO_3_ (Table 2). Similar findings were reported by the authors of [53], who noted that AgNPs produce a higher TPC. Our results indicated that AgNO_3_ negatively affected all the plant aspects toward their survival by increasing uptake and accumulation of Ag from AgNO_3_.

## 5. Conclusions

A recent study revealed that AgNO_3_ possessed more damaging effects on the growth of *P. glaucum* seedlings. However, AgNPs even at high concentrations did not severely affect the morphology and physiology of seedlings, unlike AgNO_3_. High SOD activity and a greater MDA content reflect the greater accumulation of ROS, which leads to higher membrane damage and blockage of the metabolic pathway of a cell. Thus, from the present study, it can be clearly concluded that fast and bulk release of Ag^+^ from AgNO_3_ and AgNPs causes a strong interaction with roots and also distributes to upper parts, which cause severe stress in the seedlings. Our results indicate that AgNO_3_ caused a greater reduction in total chlorophyll, carotenoid, and total protein content, which led to a greater loss of yielding due to high toxicity, whereas smaller-sized metallic AgNPs also had rapid interactions with plants and reduced the growth by impairing plant normal metabolism, but their toxic effects were limited on *P. glaucum* seedlings.

## Figures and Tables

**Table 1:** Effect of AgNPs and AgNO_3_ on root length (RL) (cm), shoot length (SL) (cm), fresh weight (FW) (g), dry weight (DW) (g), and relative water contents (RWC) (%) of pearl millet (*Pennisetum glaucum* L.) seedlings. Each experiment was performed twice with three replicates (*n* = 6). Means and standard errors (±) were calculated by analysis of variance (one-way). A comparison of the means (control + treatments) was confirmed by Duncan’s multiple range test at *p* < 0.05.

Treatments	AgNPs					AgNO_3_				
	Growth Parameters									
	RL	SL	FW	DW	RWC	RL	SL	FW	DW	RWC
Control	12.37 ± 0.96 ^c^	7.47 ± 0.23 ^c^	0.131 ± 0.006 ^c^	0.020 ± 0.0005 ^d^	95.17 ± 0.79 ^d^	12.37 ± 0.96 ^c^	7.47 ± 0.23 ^c^	0.131 ± 0.006 ^c^	0.020 ± 0.0005 ^d^	95.17 ± 0.79 ^d^
2 mM	8.87 ± 0.99 (28) ^b^	6.33 ± 0.26 (15) ^b^	0.112 ± 0.003 (14) ^b^	0.018 ± 0.0001(10) ^c^	90.01 ± 1.47 (10) ^c^	7.27 ± 0.62 (41) ^b^	5.87 ± 0.22 ^c^ (21) ^b^	0.081 ± 0.003 (38) ^b^	0.0164 ± 0.0001 (20) ^c^	83.02 ± 1.68 (13) ^c^
4 mM	7.20 ± 0.45 (41) ^bc^	5.90 ± 0.23 (21) ^b^	0.075 ± 0.002 (43) ^a^	0.016 ± 0.0002 (20) ^b^	80.54 ± 1.83 (15) ^b^	5.17 ± 0.33 (58) ^a^	5.10 ± 0.20^c^ (32) ^a^	0.068 ± 0.001 (48) ^ab^	0.0143 ± 0.0002 ^c^ (30) ^b^	72.04 ± 2.16 (24) ^b^
6 Mm	4.80 ± 0.35 (61) ^a^	5.13 ± 0.18 (31) ^a^	0.069 ± 0.002 (47) ^a^	0.0132 ± 0.0002 (35)^a^	70.28 ± 1.59 (26) ^a^	3.90 ± 0.21 (68) ^a^	4.77 ± 0.14 ^c^ (36) ^a^	0.058 ± 0.002(55) ^a^	0.0114 ± 0.0003 ^c^ (45)^a^	63.10 ± 1.49 (28) ^a^

Similar superscript letters such as (^a^, ^b^, ^c^ and ^d^) within a column indicate that means were not significantly differe between treatments *p* < 0.05.

**Table 2:** Effect of silver nanoparticles (AgNPs) and silver nitrate (AgNO_3_) on non-enzymatic antioxidants such as proline content (µg/mg fresh weight), total flavonoid content (µg/mg fresh weight), and total phenolic content (µg/mg fresh weight) of pearl millet (*Pennisetum glaucum* L.) seedlings. Experiments were performed twice with three replicates (*n* = 6). Means and standard errors (±) were calculated by analysis of variance (one-way). A comparison of the means (control + treatments) was confirmed by Duncan’s multiple range test at *p* < 0.05.

Treatment	AgNPs			AgNO_3_		
	Non-Enzymatic Antioxidants					
	Proline	TFCs	TPCs	Proline	TFCs	TPCs
Control	1.22 ± 0.037 ^a^	2.06 ± 0.043 (41) ^d^	1.56 ± 0.032 ^a^	1.22 ± 0.037 ^a^	0.59 ± 0.023 ^a^	1.22 ± 0.023 (52) ^d^
2 mM	1.59 ± 0.027 (23) ^b^	0.71 ± 0.022 (17) ^b^	1.75 ± 0.021 (11) ^b^	1.72 ± 0.032 (29) ^b^	0.84 ± 0.015 (30) ^b^	1.84 ± 0.021(15) ^b^
4 Mm	1.83 ± 0.026 (33) ^c^	0.85 ± 0.020 (30) ^c^	1.92 ± 0.024 (19) ^c^	1.93 ± 0.026 (37) ^c^	0.94 ± 0.017 (37) ^c^	2.00 ± 0.055 (22) ^c^
6 Mm	2.06 ± 0.043 (41) ^d^	1.13 ± 0.023 (48) ^d^	2.32 ± 0.028 (33) ^d^	2.16 ± 0.023 (44) ^d^	1.22 ± 0.023 (52) ^d^	2.53 ± 0.026 (38) ^d^

Similar superscript letters such as (^a^, ^b^, ^c^ and ^d^) within a column indicate that means were not significantly differe between treatments *p* < 0.05
